# Evaluation of the Oil-Rich Waste Fillers’ Influence on the Tribological Properties of Polylactide-Based Composites

**DOI:** 10.3390/ma15031237

**Published:** 2022-02-07

**Authors:** Olga Mysiukiewicz, Joanna Sulej-Chojnacka, Mateusz Kotkowiak, Tomasz Wiśniewski, Adam Piasecki, Mateusz Barczewski

**Affiliations:** 1 Institute of Materials Technology, Faculty of Mechanical Engineering, Poznan University of Technology, Piotrowo 3, 61-139 Poznan, Poland; mateusz.barczewski@put.poznan.pl; 2 Łukasiewicz Research Network—Metal Forming Institute, Jana Pawla II 14, 61-139 Poznan, Poland; joanna.chojnacka@inop.poznan.pl (J.S.-C.); tomasz.wisniewski@inop.poznan.pl (T.W.); 3 Institute of Materials Science and Engineering, Faculty of Materials Engineering and Technical Physics, Poznan University of Technology, pl. M. Skłodowskiej-Curie 5, 60-965 Poznan, Poland; mateusz.kotkowiak@put.poznan.pl (M.K.); adam.piasecki@put.poznan.pl (A.P.)

**Keywords:** polylactide, composite, tribology, waste filler, linseed oil

## Abstract

In recent years, natural-based polymeric composites have gained the attention of researchers and the industry due to their low environmental impact and good applicational properties. A promising example of these materials is polylactide-based composites filled with linseed cake. Even though they can be characterized by reduced brittleness and enhanced crystallization rate, their applicational potential cannot be fully evaluated without knowing their tribological properties. This paper is aimed to analyze the influence of the oil contained by the filler on the mechanical and frictional properties of polylactide-based composites. Specimens of unfilled polylactide and its composites containing 10 wt % of linseed cake with different oil content were prepared by injection molding. Their microhardness was measured by the Vickers method. The softening temperature was determined by the Vicat method. The scratch resistance of the samples was tested with the loading of 10, 20 and 40 N. The coefficient of friction was evaluated by the pin-on-plate method, using CoCrMo alloy as the counter surface. It was found that the oil content in the filler does not directly influence the mechanical and tribological properties, but the composite samples present comparable hardness and lower coefficient of friction than the unfilled polymer, so they can be a good eco-friendly alternative to the unfilled polylactide when the frictional properties are an important factor.

## 1. Introduction

Polylactide or poly(lactic acid) (PLA) is an aliphatic polyester synthesized from renewable resources, which can be subjected to biodegradation in industrial composting conditions [1]. Even though the procedure of its synthesis was developed by Carothers et al. in 1932 and patented by Du Pont in 1954, its high-scale production started in the 1990s [2]. The researchers, manufacturers, and consumers alike have appreciated PLA for its relative environmental friendliness [3], good mechanical properties [4], processability [5], and various modification possibilities [6]; therefore, its market has been dynamically expanding. Polylactide can replace conventional, petroleum-based plastics in the production of consumer goods and be used in special medical applications, e.g., tissue engineering [4]. PLA-based filaments are also widely utilized in industrial or home fused deposition modeling (FDM) 3D printing [7]. As polylactide in its unfilled and unmodified form is characterized by low crystallinity and crystallization rate [8], low thermal stability [9], and brittleness [10], various fillers or modifying agents are commonly used to improve its properties and widen the application possibilities [11]. Additives of almost all types, from nanometric particles [12] to micrometric fibers [13], from functionalized compounds synthesized for special applications [14] to barely processed plant-based components [15] have been successfully applied in PLA, as it is reported in numerous scientific papers. However, from the environmental point of view, the most beneficial procedure is the utilization of the so-called waste fillers, mainly the by-products from different branches of industry such as agriculture or food production [16].

Linseed cake (LC) is the residue obtained during the extraction of natural oil from flax seeds (*Linum usitatissimum* L.), which contains lignin, holocellulose, saccharides, proteins, and up to 40 wt % of the natural oil [17]. As a result of the presence of various components such as rigid lignocellulosic particles and the oil fraction, LC is an effective filler for PLA. As it was shown in our previous studies, the linseed oil contained in this waste filler has the most significant impact on the properties of the resulting composites, improving the crystallization rate [18], reducing the brittleness of the material [19], and changing its sub-zero mechanical properties [20]. The LC-filled polylactide composites are complex multiphase materials that are gaining ground in scientific research and potential high-quality polymeric composites, which can be used in various industrial applications, but the tribological properties such as friction, wear, and hardness of the composites modified with linseed cake is still an obscure area. The scope of research is even more interesting and necessary to verify experimentally, considering the potential impact of the increased content of oil migrating from the filler. Considering that friction and wear cause severe annual financial losses in the industry [21], this topic needs further research.

Friction is a force that opposes the movement of two bodies sliding against each other. It results from various phenomena in the contact objects’ surface layers, such as their adhesion and deformation [22]. As a result of their viscoelastic nature, the tribological properties of polymers are sensitive to the conditions such as sliding speed, load, external temperature, and testing time [23]. Therefore, there can be discrepancies between the results obtained in different studies, but some general ideas about the wear and friction of polylactide can be found in the literature. As revealed from the literature research, the coefficient of friction of unmodified PLA was studied for the first time by Rafael Auras in 2004, who determined its value at 0.32–0.37 [24]. The first widely available study of the tribological properties of polylactide-based composites was published nine years later by Bajpai, Singh, and Madaan [25]. They found that the coefficient of friction depends on the testing conditions (such as the applied load and sliding speed) as well as the interactions between the matrix and the filler. The majority of the most current studies about the tribological performance of PLA-based materials focus on the FDM-printed specimens [26]. The polylactide composites containing natural waste fillers remain a mostly obscure area, which needs research. The tribology of linseed cake-filled PLA is an especially interesting subject because of the presence of natural oil. As it was found by Quinchia et al., different vegetable oils can serve as lubricants in a wide range of conditions [27]. As Myshkin, Grigoriev, and Kavaliova found out, linseed oil presents even better tribological characteristics than a base mineral oil [28]. The advantageous tribological properties of different plant oils modified with boric acid were also shown by Trzepieciński in their paper [29]. To summarize, while the mechanisms of the influence of various types of natural oils on the tribological properties of polymers have been considered before [14,28,29], no description of the influence of complex modification phenomena induced by the incorporation of fillers with high oil content on tribological properties of thermoplastic composites has been reported so far. In this case, the influence of oil on changes in the wear of composite samples will result from both polymeric structural changes caused by the presence of lignocellulosic filler and the effects of oil migration. Based on the previously conducted research [30], a question should be asked as to whether the increasing share of oil in the fillers will allow classifying composites modified with natural waste from the food industry as functional fillers showing a beneficial effect on tribological properties.

The research results have shown the compatibility of various natural oils, including linseed oil, with the polylactide matrix [30]. The oil was established in the interphase in the form of micro-scale domains and at the level of macromolecular dispersion; however, there was no effect of oil exudation from polymer samples. Therefore, it can be hypothesized that the natural linseed oil contained by the waste filler could act as an internal lubricant and improve the frictional characteristics of PLA, leading to obtaining self-lubricating sustainable composites. This paper aims to analyze the influence of the oil content in the linseed cake on the tribological properties of PLA-based composites, including coefficient of friction, microhardness, scratch resistance, and wear.

## 2. Materials and Methods

### 2.1. Materials

Linseed cake was purchased from a local supplier and fractioned using an Analysette sieve shaker (Fritsch, Weimar, Germany) equipped with a 630 μm mesh. To investigate the influence of linseed oil on the tribological properties of the composites, the filler was subjected to partial defatting in acetone. As a result of the procedure described in our previous study [19], 5 grades of linseed cake with 0.9, 4.6, 17.7, 30.4 and 39.8 wt % of natural oil were obtained and used to produce the composites.

A multipurpose PLA grade Ingeo 2500HP (Natureworks, Minnetonka, MN, USA) characterized by a density of 1.24 g/cm^3^ and melt flow index of 8 g/10 min (210 °C, 2.16 kg) was chosen as the matrix of the composites.

### 2.2. Composite Preparation

The filler and the polymer were dried at 70 °C overnight in a laboratory cabinet drier before each processing step. The samples containing 10 wt % of the filler were obtained by the melt mixing method using a Zamak EHD 16.2 co-rotating twin-screw extruder (Zamak, Skawina, Poland) with processing parameters of 100 rpm screw rotational speed and maximum temperature set of 190 °C. After mixing in a molten state, the extrudates were pelletized and formed by injection molding using a Battenfeld PLUS-35 machine (Battenfeld, Kottingbrunn, Austria). The injection temperature of 210 °C, the mold temperature of 50 °C, and the injection pressure of 72 MPa were applied. The specimens of unfilled PLA were prepared in the same way as the composites. A more detailed description of the samples’ extrusion and injection molding can also be found in our previous paper [19]. The samples were named in reference to the oil content in the filler, e.g., the name PLA-LC4.6 indicates the polylactide-based composite filled with 10 wt % of the linseed cake containing 4.6 wt % of linseed oil.

#### Pretreatment of the Samples for the Frictional Measurements

**Figure 1:**
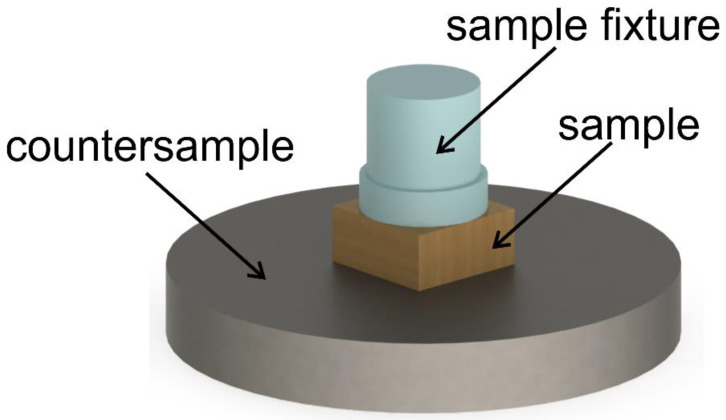
Friction test setup—a composite sample in the resin fixture which was needed to mount it in the apparatus.

The injection-molded polymeric samples were cut into 9 mm rectangles and polished by hand with 2000 grid sandpaper, so the resulting surface roughness Ra was approximately 0.5 μm. To mount them in the testing machine, a dedicated resin fixture had to be prepared. The samples were placed in silicone molds and filled with a 100:28 mixture of the Epidian CHS-EPODUR 574–0512 A epoxy resin with the CHSE 574–0512 B hardener (Ciech, Nowa Sarzyna, Poland). The specimens were cured for 48 h at room temperature and then post-cured at 80 °C for 4 h. Prior to the measurement, the tested surface was cleaned with ethanol. The specimen used in the test is schematically shown in Figure 1.

### 2.3. Methods

#### 2.3.1. Microhardness

Microhardness of PLA and its composites was tested by the Vickers method using the Micromet II tester (Buehler, Uzwil, Switzerland). The load time was set to 15 s. The test load of 50 g was applied. The mean value and the standard deviation for each material were calculated from at least 8 separate measurements.

#### 2.3.2. Vicat Softening Temperature

The Vicat softening temperature (VST) of the studied materials was determined according to the ISO 306 standard using an HDT/Vicat testing apparatus RV300C (Testlab, Warsaw, Poland). A loading of 10 N and heating rate of 120 °C/h were applied.

#### 2.3.3. Scratch Resistance

The scratch resistance of the samples was evaluated using a Line Art 249 hardness tester (Erichsen, Hemer, Germany) equipped with a tip of 0.6 mm. During the test, the speed of the tip was set to 35 mm/s. Samples of each kind were tested with the load of 10 N, 20 N and 40 N. The scratch width was measured using the optical microscope SMZ-143 (Motic, Hong Kong) and the Images Plus 2.0 software (Motic, Hong Kong). The scratches created under the 10 N loading and, in the case of unmodified PLA, 20 N loading were blackened with a marker pen to make them more visible. The mean scratch width and standard deviation were calculated for each test based on 15 individual measurements.

#### 2.3.4. Scanning Electron Microscopy

The scanning electron microscope (SEM) Tescan MIRA3 (Tescan, Brno, Czech Republic) was used to assess the surface after tribological evaluation of the PLA and its composites. The worn surfaces of the tested samples were assessed with an accelerating voltage of 12 kV and a working distance 16 mm. The thin carbon coating with a thickness of approximately 20 nm was deposited on samples using the Jeol JEE 4B vacuum evaporator.

#### 2.3.5. Coefficient of Friction

Measurements of the coefficient of friction were performed in a pin-on-plate configuration using a T-17 apparatus manufactured by Institute for Sustainable Technologies—National Research Institute (Radom, Poland). A disc of a CoCrMo alloy characterized by surface roughness Ra of 0.01 μm was used as the counter surface during the test. It was chosen because of its corrosion resistance, so it does not react with the polymeric samples, and the results obtained for the composites with different fillers could be compared.

At least three separate measurements were performed for each material. Averaged and smoothed curves of coefficient of friction vs. time were prepared using Origin software.

The measurements were conducted in the following conditions: amplitude of the reciprocating movement: 6 mm, frequency of 10 Hz, a load (*F_n_*) of 10 N, and testing time of 4 h. The friction force *T* was recorded during the test as a function of time, and the coefficient of friction *μ* was calculated according to Formula (1):

(1) $$\mu =\frac{T}{F_{n}}.$$

The worn surfaces were observed using an Opta Tech SK stereoscopic microscope equipped with RT 16 Mpx digital camera (Opta Tech, Warsaw, Poland).

The specific wear rate *W_s_* of the samples subjected to the friction test was calculated according to Formula (2)

(2) $$W_{s}=\frac{∆V}{F_{n}\cdot D}$$

where Δ*V*—the volume difference (mass loss/sample density) (mm^3^), *F_n_*—load, *F_n_* = 10 N, *D*—sliding distance, *D* = 432 m.

## 3. Results

### 3.1. Characteristics of the Materials

The most important mechanical, thermal, and thermomechanical properties of PLA and linseed-cake composites are collected in Table 1. It can be observed that the addition of the oil-rich filler to polylactide makes the composites less stiff, more prone to plastic deformation, and resistant to impact fracture. The LC-filled samples also undergo glass transition at a lower temperature and achieve a higher degree of crystallinity in comparison to the unmodified polymer. These results can be explained by the plasticizing influence of the oil contained in the filler, which enhances movement possibilities of macromolecules, facilitating the relaxation and the formation of the crystalline phase during cooling from the melt. The presence of the plasticizing filler prevents brittle fractures during quasi-static and dynamic loading. A much more in-depth analysis of the influence of the oil contained by linseed cake on mechanical, thermal, thermomechanical, and structural properties of polylactide-based composites can be found in our previous studies [18,19,31].

### 3.2. Microhardness

**Figure 2:**
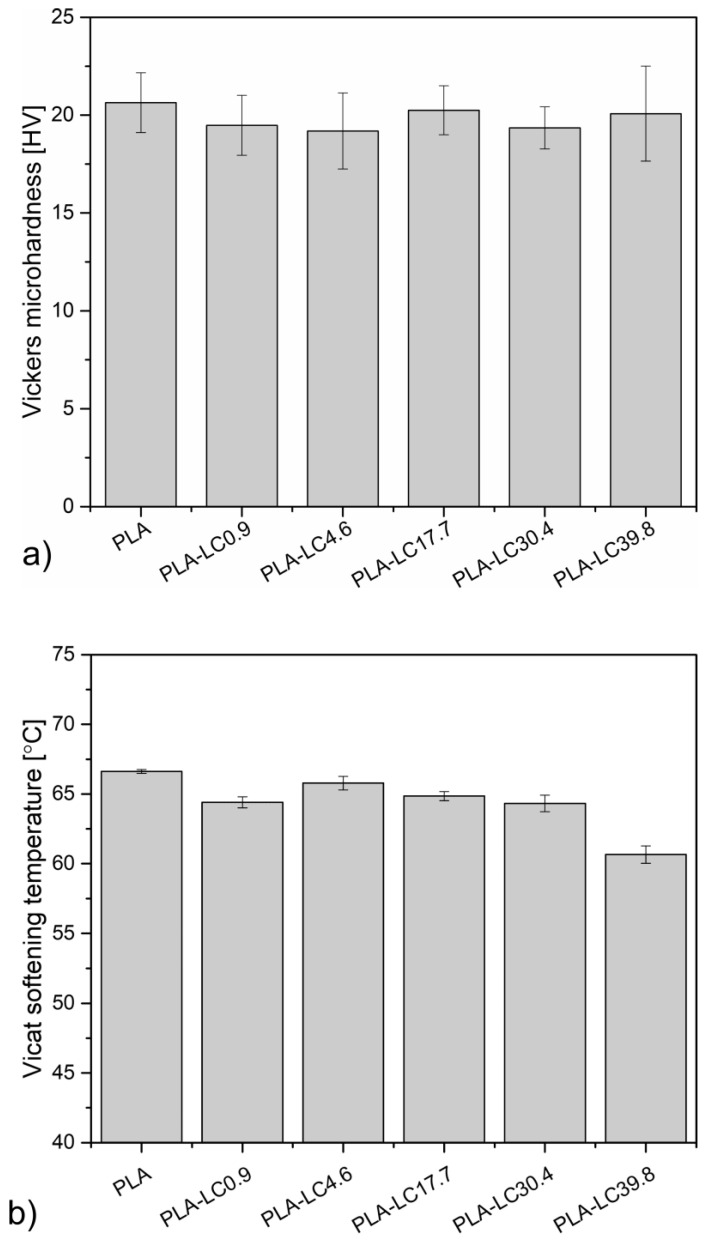
Vickers microhardness (**a**) and Vicat softening temperature (**b**) of PLA and its composites.

The Vickers microhardness of PLA and linseed cake-modified composites is presented in Figure 2a. The unfilled polylactide can be characterized by a microhardness of about 20.6 HV, which is a commonly observed value [32]. The composite samples present slightly lower values in the range of 19.2–20.3 HV. There is no clear relationship between the specimen’s composition and its microhardness—the lowest value was measured for PLA-LC4.6 and the highest (except for the unmodified PLA) was measured for the PLA-LC17.7. This behavior is understandable, as the microhardness of a composite material results from multiple factors such as the mechanical properties of the phases [33], the presence of plasticizers [34], the crystallinity of the polymeric matrix, and the interactions of the filler and the polymer [35]. The slight decrease in the microhardness can be attributed to the presence of lignocellulosic particles, which are softer than PLA [33], and to the influence of the linseed oil, which has a plasticizing effect on the polymeric matrix [36]. On the other hand, the presence of the natural oil simultaneously improves PLA’s ability to crystallize [18], which should increase the hardness of the material. As multiple factors influence the microhardness of the material, which counteract each other, the final value remains comparable. Similar results were reported by Agüero et al. in the case of PLA modified by flaxseed flour and modified linseed oil—the hardness of the composites also remained constant, regardless of their content [37].

Considering the possible occurrence of thermal effects induced by friction during the tribological measurements [38], it is essential to evaluate the changes in the softening temperature of the various composite materials in order to exclude the risk of not taking into account additional factors on measured data. The Vicat softening temperature (VST) allows evaluating the thermomechanical properties of a materials’ surface, as it depends on both the hardness and the glass transition. Its values obtained for PLA and the composites are presented in Figure 2b. Unfilled polylactide softens at about 66.6 °C, which is associated with this glass transition taking place around this temperature [39]. The addition of linseed cake causes a decrease in the VST, which is especially notable in the case of the PLA-LC39.8 sample. As the addition of the rigid plant-originated fillers such as buckwheat husk or wood usually causes an increase in the VST values [9,10], this result should be connected as an effect of the linseed oil present in the filler. As shown by Balart et al., the addition of epoxidized linseed oil caused a decrease in the polylactide’s softening temperature, which was attributed to the plasticizing effect of the additive [36]. Similar results were reported in the case of PLA plasticized by octyl epoxy stearate [40]. The described differences between the unfilled polymer and its composites are less pronounced than in the cases described in the cited literature, which can be attributed to the stiffening effect of the rigid lignocellulosic fraction, which limits the softening effect of the oil. Based on the VTS and microhardness analysis, it can be stated that the oil contained by the filler acts as a plasticizer and facilitates the movement of the polylactide macromolecules, but its influence is much more pronounced at elevated temperatures.

### 3.3. Scratch Test

**Figure 3:**
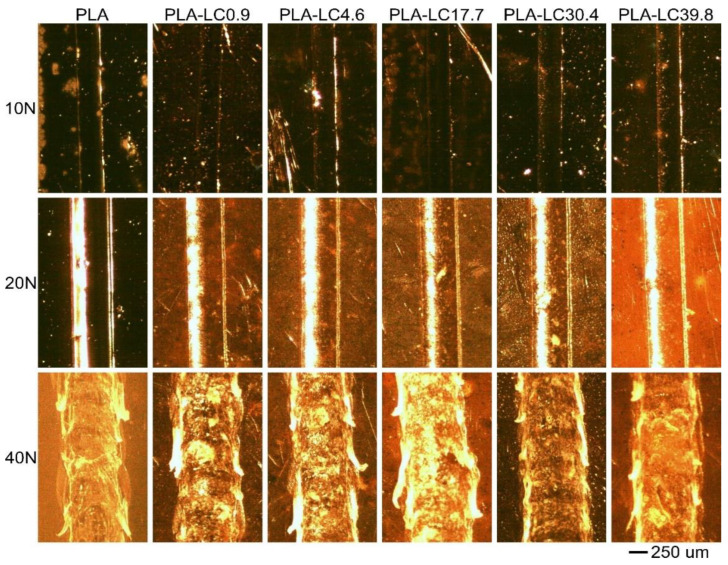
Microscopic images of scratches created on the samples during the scratch resistance evaluation.

**Figure 4:**
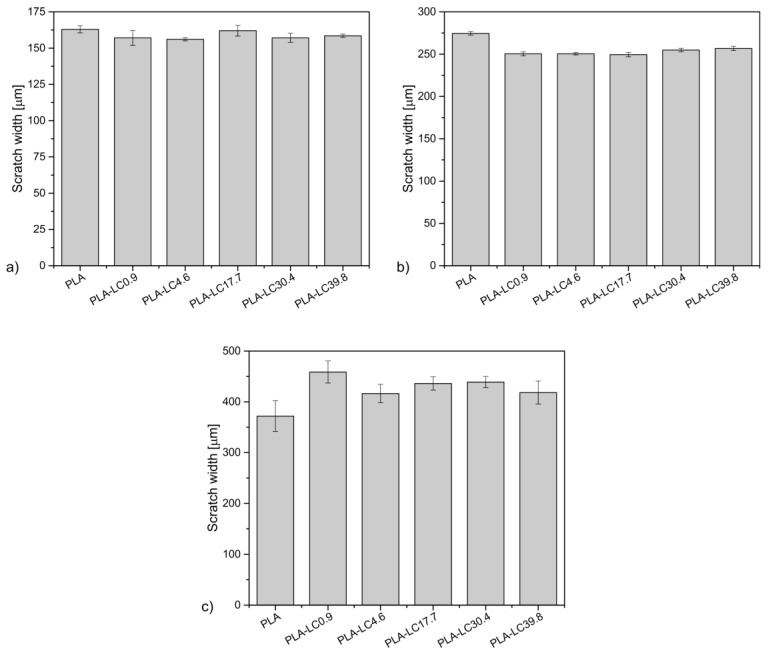
Scratch width created on samples under the load of 10 N (**a**), 20 N (**b**), and 40 N (**c**).

The optical microscope images of the scratches are shown in Figure 3. Their mean width is presented in Figure 4. The damage resulting from the scratch test procedure can be typically ascribed to one of three stages: invisible damage (smooth indentation) under the lowest loading (Stage I), groove formation under the intermediate loading (Stage II), and finally, material removal (plowing) under the highest loading (Stage III) [41]. As it can be seen in Figure 3, under the load of 10 N, all the studied materials present Stage I damage, which is indicated by smooth, shallow indentation. Its width is in the range of 156–163 μm, and similar values were recorded for PLA and its composites with different filler content. It can be presumed that in this stage, the deformation only occurs in the outermost layer of the sample, which in the composite samples mainly consists of the matrix material [42], so the presence of the additives does not influence the resulting scratch. Different behavior can be observed under the loading of 20 N—the visibly more pronounced grooves shown in Figure 3 can be ascribed to Stage II, which indicates plastic deformation of the material. The width of the scratch created on unfilled PLA is 275 μm and 249–257 μm for the composite samples. The thinnest grooves (249 and 250 μm) can be observed on the surfaces of the specimens containing up to 17.7 wt % of the linseed oil. For the oil-rich samples, the scratches are slightly wider. It can be assumed that the presence of the defatted linseed cake particles limits the movement possibilities of the polymeric chains, so the composites are more resistant to plastic deformation during scratching. However, in the case of the materials containing a considerable amount of linseed oil with the plasticizing ability, the polymeric matrix can deform more easily because of the enhanced mobility of the macromolecules. The potential for oil domains to accumulate in the polymer–filler interface for the fillers with the highest oil concentration should also be considered [30]. This phenomenon can cause a reduction in adhesion between the filler and the polymer, which will result in additional pull-out effects in the contact area. Simultaneously, the presence of the natural oil causes an increase in the material crystallinity [18], which improves the scratch resistance of the polymeric material [41]. As an effect of the two phenomena, the PLA-LC30.4 and PLA-LC39.8 specimens present better resistance to Stage II scratch than the unfilled polylactide but not as good as the composites containing the partially defatted filler.

The differences in the scratch resistance of the studied materials are lower when the 40 N load is applied. The Stage III damage, which is material removal caused by the excessive tangential force [43], can be observed both for PLA and its composites. The naked eye can see the uneven scratches and can be characterized by a width of 372–459 μm. Unlike in the 20 N loading case, the thinnest scratch was measured for the unfilled PLA, and the widest was measured for the PLA-LC0.9 sample, whereas the remaining composite samples present the scratch width of 416–438 μm independently of their composition. This divergence can be explained by a different damage mechanism—in Stage III, the continuity of the material needs to be broken. The material removal is easier in the multiphase samples when the adhesion between the filler and the matrix is insufficient. Therefore, the scratch width increases in the composites containing the hydrophobic polylactide [44] and hydrophilic linseed cake [45]. It can be concluded that the scratch resistance of the linseed cake-filled PLA composites depends on the loading and the addition of the oil-rich waste filler. The effect is most advantageous when the material is subjected to intermediate loading—in Stage I mode, the depth of the scratch is so small that it only affects the outermost polymeric layer of the sample and in Stage III, plowing of the material is easier because of the low affinity between the phases. However, in the plastic deformation conditions, the filler limits the movement possibilities of the polymeric chains, increasing the resistance of the composite. Even though the linseed oil acts as a plasticizer in PLA, which could make the material more susceptible to Stage II damage, it also improves the crystallinity of the polymeric matrix, which counteracts the decrease in scratch resistance [41].

### 3.4. Coefficient of Friction

**Figure 5:**
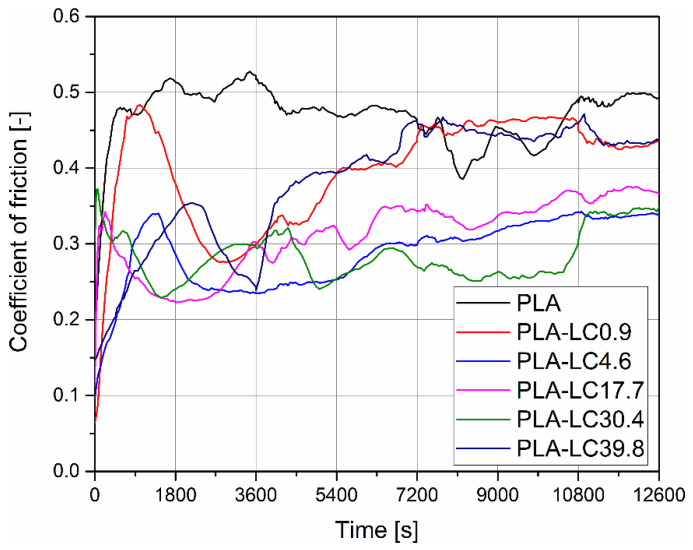
The changes of the coefficient of friction of PLA and its composites during the test.

The changes of the coefficient of friction (*μ*) during the pin-on-plate test are presented for PLA and its composites in Figure 5. In the first stage of the test, the value of *μ* increases rapidly to a maximum. As it is generally known, the frictional force *F* depends on the real contact area *A_r_* between the two bodies and the effective shear strength of contacts *τ* in a way described by Formula 3 [23]:

(3) $$F=A_{r}\cdot \tau .$$

During the run-in period, only the peaks of the asperities present on the surfaces of the tribological pair come into contact, and the resulting real contact area is relatively small, which results in low frictional force. When the asperities are sheared, the *A_r_* increases, resulting in the rapid growth of the coefficient of friction. For the unfilled PLA, it takes about 570 s (9.5 min) to reach a maximum *μ* of 0.48. In the case of all the composite samples apart from PLA-LC0.9 (which, in this stage of the test, behaves similarly to the unmodified polylactide), the value of the first peak of the coefficient of friction is in the range of 0.32 to 0.37. This discrepancy can be explained by the lower shear strength of the linseed oil-modified polymer. The asperities present in the surface of the samples are easier to even out, which, according to Formula 3, results in lower shear strength.

During the tribological test performed in constant conditions such as sliding speed and load, polymeric materials tend to achieve a steady state, when the value of the coefficient of friction oscillates around a constant value [23]. However, even in the case of the unfilled polymer, the coefficient of friction fluctuates during the remaining part of the test. Interestingly, the steady state is typically not achieved in published studies on the tribological properties of polylactide-based materials [46]. For example, in the case of 3D-printed PLA, fluctuation of the coefficient of friction was explained by elastic recovery of the material [47]. Hanon et al. connected this behavior to the slip-stick phenomenon or transfer of the material on the counter sample [48]. Another possible factor is the softening of PLA due to a local increase in temperature, which changes the type of contact between the two bodies [49]. The latter explanation of the lack of the steady state observed in the case of polylactide-based materials is especially interesting because of the complex thermal properties of this polymer, which undergoes glass transition and cold crystallization in a relatively small range of temperatures, and each of these changes can influence the tribological performance of this polymer.

The linseed cake-filled samples also do not achieve the steady state under the testing conditions, but their behavior is different: After the first maximum attributed to the run-in period, the *μ* value decreases visibly. For example, the PLA-LC0.9 shows a drop of the coefficient of friction from 0.48 to 0.28, and PLA-LC39.8 shows a drop from 0.35 to 0.24. This decrease can be attributed to the formation of the transfer film, which acts as a lubricating agent [50]. It may be presumed that in the composite samples, because of their more complex structure and lower shear strength, the transfer of the material to the counter surface is easier, and the transfer film creation is more effective. What is more, the presence of the filler is also known to facilitate the formation of the transfer film. The addition of micro-sized fillers such as Ni or Al_2_O_3_ particles is known to result in the creation of more uniform and thinner transfer film, which results in a reduction of wear [51]. Even though the linseed cake particles are larger than most of the studied fillers intended for tribological applications, they are also relatively softer (in comparison with metallic or ceramic materials), so they do not cause excessive ploughing of the polymeric surface. Moreover, as it was shown by the analysis of the Vicat softening temperature, the linseed oil contained by the filler has a softening influence on polylactide. During the frictional test, the sample’s surface is locally heated due to the dissipated energy. In these conditions, the influence of the linseed oil is intensified, it reduces the intermolecular attraction forces between the macromolecules of PLA [36], so they can create the transfer film more easily and efficiently. During the remaining part of the test, after the decrease in the coefficient of friction, the *μ* rises again, but the increase is not steady. This complex behavior can be explained by several simultaneous phenomena, such as destruction of the transfer film [51], debonding of the filler particles from the polymeric matrix [52], deposition of the wear products in the wear track [47], plowing of the polymeric surface by the metallic counter surface [53], and also shearing of the asperities [54].

Analyzing the coefficient of friction of PLA and its composites, it can be seen that the *μ* value of the linseed cake-filled materials is almost in the entire course of the test lower than in the case of the unmodified polymer. However, there is no direct relationship between the filler’s oil content and the frictional properties—at the final stage of the test, the PLA-LC0.9 and PLA-LC39.8 present almost the same values of *μ*. Therefore, it can be concluded that the natural oil present in the filler does not act as an internal lubricant in the measurement conditions. Even though the linseed oil and polylactide miscibility is limited and the excess of the oil creates droplet-like domains in the polymeric matrix [19], it does not leach out of the composite material to the sample’s surface during the test. In fact, the performed tribological test helped reveal useful information about the internal structure of the studied composites: it was confirmed that the oil contained by the filler does not migrate to the sample’s surface. What is more, the frictional properties of polymeric materials are strongly linked to their mechanical properties, such as hardness [55]. As we have already shown in the microhardness evaluation and scratch resistance analysis, there is no direct influence of the linseed oil content on these parameters; therefore, the tribological properties of different composites are also similar. Finally, the influence of the counter sample on the results of the tribological measurements cannot be omitted. As it was shown by Menezes et al., the coefficient of friction is controlled by the more rigid counter surface [53], and the ratio of the hardness of different PLA-based composites and CoCrMo alloy is similar, regardless of the type of the linseed cake. From the application point of view, it can be concluded that the LC-filled composites show similar or even better tribological properties compared to PLA, which is a beneficial result.

**Figure 6:**
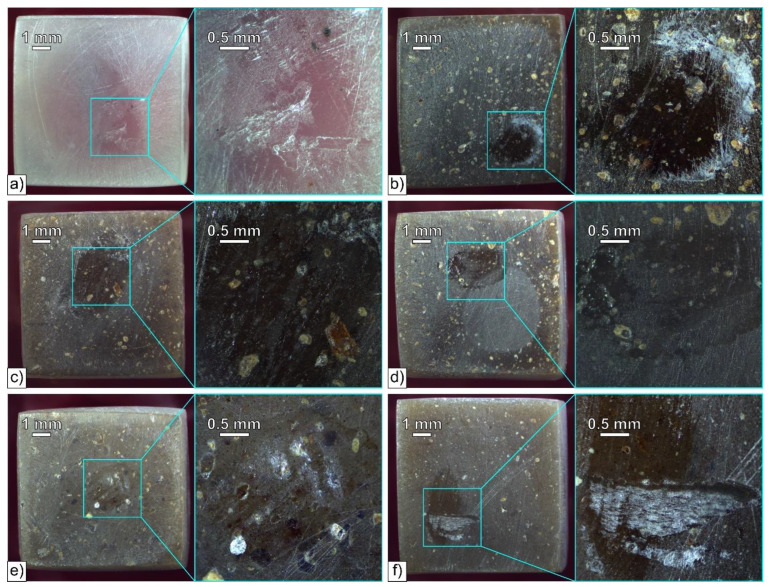
Microscopic images of the worn surfaces of PLA (**a**), PLA-LC0.9 (**b**), PLA-LC4.6 (**c**), PLA-LC17.7 (**d**), PLA-LC30.4 (**e**), and PLA-LC39.8 (**f**).

The microscopic images of the worn surfaces are shown in Figure 6. All the studied specimens look similar: apart from scratches and grooves present on the entire worn surface, smaller “polished” regions are visible in the middle of the samples, which are marked with blue squares and magnified in Figure 6. Accumulated debris can be seen next to the smooth regions, which indicates that the wear products polished them. It is also possible that despite precise polishing of the samples before the test, the actual contact area was smaller than the entire sample’s surface, which was presumably due to the plastic deformation of the sample in the holder of the T-17 apparatus. The filler particles can be easily seen on the worn surface in the composite samples, which are accompanied by cracks on the filler–matrix interface. Debonding of several linseed cake particles also took place, but most of them are still embedded in the polymeric matrix. As it can be seen in Figure 6, the wear resistance of the PLA-based composites is not severely influenced by the addition of linseed cake.

**Figure 7:**
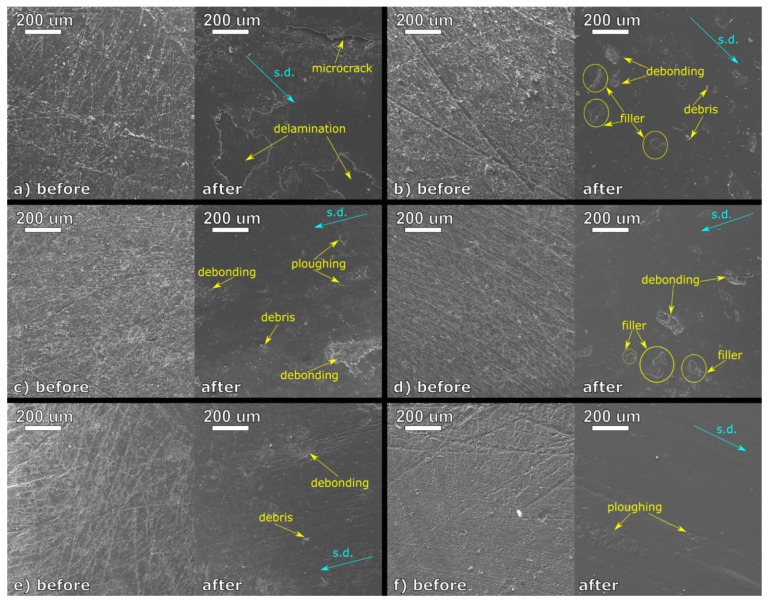
SEM images of the surfaces of the samples before and after the frictional test: PLA (**a**), PLA-LC0.9 (**b**), PLA-LC4.6 (**c**), PLA-LC17.7 (**d**), PLA-LC30.4 (**e**), and PLA-LC39.8 (**f**). S.d. indicates the sliding direction.

In order to evaluate the wear behavior of PLA and the composites made using oil-rich filler, SEM images of their surfaces before and after the frictional testing were obtained and presented in Figure 7. As it can be seen, all the samples prepared for the testing have a similar appearance. They can be characterized by randomly distributed scratches resulting from grinding the specimens with sandpaper. The worn surfaces of the studied materials also reveal some standard features—the grooves and asperities resulting from the samples’ pretreatment are evened out. However, apart from the “polished” appearance, there are noticeable differences between the PLA and its composites. The unfilled polymer subjected to the frictional test reveals microcracks and delaminations on its surface. They can be attributed to the formation of the transfer film on the counter surface. However, due to the brittle nature of PLA, the transferred material is “pulled out” of the sample, as the sharp edges of the delaminations indicate it. In the case of the composite samples, filler particles can be easily observed on the worn surfaces. Some of them are still embedded in the polymeric material, but debonding of the linseed cake particles also occurs. This behavior results from limited adhesion of the hydrophilic plant-based filler and hydrophobic polymer and commonly occurs in the case of polymeric composites filled with natural substances [46]. Interestingly, the number of visible filler particles and debonding sites decreases along with the oil content in linseed cake. This phenomenon is presumably caused by fragmentation of the less stiff oil-rich filler in the polymeric matrix during melt processing at high shearing rates [18]. Plowing can also be spotted on the worn surfaces of the PLA-LC4.6 and PLA-LC39.8, which can be identified as plastic deformation of the material. Considering “brittle” delamination of the unfilled polylactide’s surface, it can be decided that this behavior results from the plasticization of the polymer by the oil-rich filler. Finally, debris (wear products such as broken transfer film or debonded particles) can also be seen in the SEM images of the worn composite surfaces, as it was also noticed during the optical microscopy observations.

The values of the specific wear rate of the samples subjected to the friction test are collected in Table 2. As it can be seen, the results obtained for all the samples do not exceed 5.2∙10^−8^ mm^3^/(Nm), which is comparable with the results obtained by Bajpai et al. for plant fiber-reinforced PLA composites [25]. Even though the lowest *W_s_* value is obtained for the unfilled polymer, its composites show comparable values. Similar to the remaining tribological properties studied in this paper, no direct correlation of the samples’ composition and the specific wear rate can be noticed, especially for the composites containing less than 17.7 wt % of oil in the filler. In the case of the oil-rich samples, their *W_s_* values decrease along with the oil content, which the improved crystallinity of these materials can cause.

## 4. Discussion

Based on all the performed measurements, the conclusions can be made about the influence of the oil contained by the filler on the tribological performance of polylactide-based composites. First, the hypothesis that linseed cake provides an internal lubricant for PLA-based materials was disproved. This result was explained by the fact that the natural oil, which is only partially miscible with the polymeric resin and forms separate domains in the polymer, does not migrate to the sample’s surface. Even though this observation can be perceived as disadvantageous from the tribological point of view, it is highly beneficial, considering the long-term performance of linseed cake-filled composites for non-tribological applications.

Although most of the linseed oil remains in its domains during the tribological test, a small fraction that is miscible with the polymeric material influences its behavior. Based on the SEM observations of the worn surfaces, the LC-filled samples deform in a plastic way, in contrast to the brittle fractures seen on the unfilled polylactide’s surface. It can be explained by the plasticizing effect of the oil, which facilitates the movements of the macromolecules. This effect is much more pronounced in elevated temperatures, as indicated by the decrease in the Vicat softening temperatures observed for the oil-rich samples. However, when friction takes place at room conditions and only a local increase in temperature is observed, the presence of the filler does not significantly change the tribological behavior of the studied composites.

## 5. Conclusions

The tribological and mechanical properties of polylactide-based composites were successfully evaluated. It was found that even though the natural oil present in the linseed cake influences the structure of the PLA composites, it does not have a significant influence on the microhardness, which remains stable around 20 HV, regardless of the used LC type. The influence of the filler on the scratch resistance of the studied samples is more complex and depends on the mode of deformation and the value of the loading. When the composites are subjected to plastic deformation, the presence of the filler particles improves their resistance to scratching. The most interesting results were obtained in the case of the frictional properties. Although the linseed oil contained by the filler does not act as an internal lubricant in polylactide, the composite samples present lower coefficient of friction values than the unfilled polymer. This result can be explained by the formation of more uniform and thinner transfer film due to the presence of the filler and lower shear strength of the composites. It was also found that the oil contained by the filler does not migrate to the surface of the sample, which is highly beneficial taking into consideration the long-term non-tribological applications of the composites. It can be also decided that polylactide filled with linseed cake, a cost-effective, sustainable waste filler, can be successfully used in industrial applications, where the tribological properties are an important factor.

## Figures and Tables

**Table 1:** Mechanical, thermal, and thermomechanical properties of PLA-based composites.

Property	PLA	PLA-LC0.9	PLA-LC4.6	PLA-LC17.7	PLA-LC30.4	PLA-LC39.8
Tensile strength [MPa] ^1^	74.3 ± 0.39	59.4 ± 0.18	56.5 ± 2.43	53.7 ± 0.64	46.4 ± 0.87	36.7 ± 0.22
Tensile modulus [MPa] ^1^	2270 ± 400	2430 ± 65	2270 ± 88	2160 ± 102	1890 ± 44	1650 ± 90
Elongation at break [%] ^1^	8.0 ± 1.80	4.5 ± 0.21	4.4 ± 0.37	4.9 ± 0.27	16.0 ± 6.0	45.0 ± 5.4
Impact strength [kJ/m^2^] ^2^	2.38 ± 0.20	1.83 ± 0.30	2.17 ± 0.40	2.34 ± 0.40	2.42 ± 0.40	3.12 ± 0.10
Glass transition [°C] ^1^	70.2	69.6	68.2	68.2	67.8	67.4
Crystallinity [%] ^1^	32.5	40.2	40.9	43.7	57.6	65.2

^1^ Measurement conditions and an in-depth interpretation of the results can be found in our previous paper [19]. ^2^ Measurement conditions and an in-depth interpretation of the results can be found in our previous paper [20].

**Table 2:** Mass loss, density, and the specific wear rate of the worn samples.

Sample	Mass Loss [10^−4^ g]	Density ^1^ [g/cm^3^]	Volume Difference [10^−4^ mm^3^]	Specific Wear Rate [10^−8^ mm^3^/(Nm)]
PLA	0.7	1.239	0.6	1.3
PLA-LC0.9	2.8	1.246	2.2	5.2
PLA-LC4.6	1.3	1.249	0.10	2.4
PLA-LC17.7	2.0	1.238	0.16	3.7
PLA-LC30.4	1.6	1.228	0.13	3.0
PLA-LC39.8	1.3	1.209	0.11	2.5

^1^ The analysis of the density of the PLA-based composites with linseed cake was presented in our previous article [19].

## Data Availability

Not applicable.
